# Design and Performance Test of Transformer Winding Optical Fibre Composite Wire Based on Raman Scattering

**DOI:** 10.3390/s19092171

**Published:** 2019-05-10

**Authors:** Yunpeng Liu, Junyi Yin, Yuan Tian, Xiaozhou Fan

**Affiliations:** 1 State Key Laboratory of Alternate Electrical Power System with Renewable Energy Sources, North China Electric Power University, Baoding 071003, China; liuyunpeng@ncepu.edu.cn (Y.L.); tianyuan_274@126.com (Y.T.); fxz@ncepu.edu.cn (X.F.); 2 Hebei Provincial Key Laboratory of Power Transmission Equipment Security Defence, North China Electric Power University, Baoding 071003, China

**Keywords:** Raman scattering, transformer winding, distributed optical fibre, temperature detection, hot-spot location

## Abstract

Winding overheating is a common fault in a transformer. To detect the temperature, the most widely used method is a point-type measurement, but traditional measurement methods cannot obtain the whole temperature distribution in a transformer. Taking this into consideration, a new method with which to measure the temperature of transformer windings was proposed. Based on Raman scattering, a new fibre-optic composite winding model was developed. The feasibility of the model was verified by electrical as well as temperature, field simulation and power frequency resistance testing. To assess the practicality and accuracy of the new model, a distributed optical fibre temperature measurement platform was built, and a series of experiments were designed. According to the data collected, the temperature measurement error based on the method could be limited to 1 °C while the positioning accuracy error was within 1 m, which meant that the new approach can satisfy the requirements of transformer winding temperature measurement and locate hot spots in the winding.

## 1. Introduction

Power transformers, as essential equipment for the operation of a power grid, play an important role in the stable transmission of power throughout the grid and in changes in voltage therein. With power systems developing into ultra-high voltage and large-scale power grids, the impact of transformer faults on the safe operation of power systems is increasing. The safe and stable operation of a transformer is of vital importance to the safety and reliability of the entire power grid. Therefore, improving the safety, reliability and service life of power transformers is a key issue in current research [1,2,3,4].

The life and operational reliability of power transformers are mainly determined by the condition of their insulation, and the most important factor affecting their insulation capability is local hot-spot temperature during operation [5]. When the transformer is in normal operation, there are energy losses in the iron core, windings and other metal parts, mainly including no-load loss and load loss. After these losses are converted into heat, the temperature rises: the temperature of the windings is the highest and the heat distribution therein is uneven. Once local overheating occurs, the insulation, and safe operation, of the transformer will be affected. Therefore, it is necessary to monitor hot spots on windings to avoid overheating.

Currently, the methods used for measuring the internal temperature of transformers primarily include top-oil temperature measurement methods [6,7,8], fibre Bragg grating (FBG) methods [9,10,11,12], fluorescence fibre measurement methods [13,14], among others. The traditional top-oil temperature measurement method has a small measurement range and low measurement accuracy; the fibre Bragg grating temperature sensor has relatively good performance, but it is a point-type measurement, and the overall distribution of the internal temperature field of the transformer cannot be obtained, and at the same time, due to the narrow internal space of the winding, the difficulty and cost of installing FBG is high. The fluorescent fibre method is still a point-type measurement, which cannot guarantee that the point measured is a hot spot, and it needs more research in terms of fibre insulation, oil aging and lifetime accuracy.

Distributed optical fibre sensing technology is a new sensing technology which has developed rapidly in recent years [15]. It utilises the low-loss transmission characteristics of optical fibres to detect physical quantities such as the temperature and strain around the optical fibre by analysing the distribution of backscattered light from the optical fibre. It is a fully distributed fibre optic sensing technology. It benefits from its small size, light weight, good temperature resistance, strong anti-electromagnetic interference, long transmission distance, and convenient telemetry and control [16,17]. The application range is very wide, including high-voltage power cables [18,19,20], ice-covered high-voltage transmission line condition monitoring, bridges, highways, other dynamic detection systems, fire alarms, etc. [21].

Therefore, using distributed optical fibres to monitor the temperature field distribution in transformer windings has the potential for broad practical application. For this study, a distributed optical fibre testing method for the temperature of transformer winding based on Raman scattering was investigated, a distributed temperature detection system was designed, and the winding temperature was measured directly by conducting temperature measurements and hot-spot positioning tests. Compared with the existing winding temperature measurement methods, the distributed optical fibre sensing technology can accurately measure the winding temperature and accurately locate hot spots, which provides a new method of monitoring the temperature around transformer winding hot spots.

## 2. Detection Principle

### 2.1. Light Scattering in Optical Fibres

**Figure 1:**
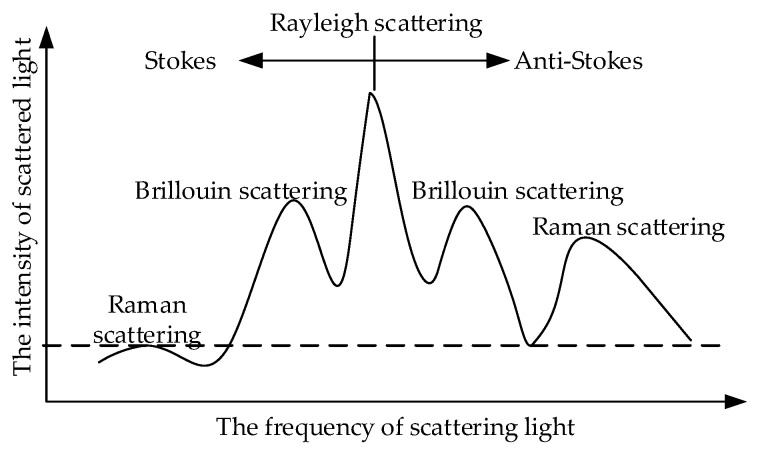
Scattering in optical fibre.

When light propagates in the optical fibre, light scattering occurs due to the unevenness of the material in the optical fibre. Scattering can be divided into elastic and inelastic scattering. The frequency of elastic scattered light is the same as the frequency of the incident light, whereas the frequency of inelastic scattered light is offset from the frequency of the incident light. The study found that the scattering of light in the fibre includes Rayleigh scattering, Brillouin scattering and Raman scattering (Figure 1) [22]. Rayleigh scattering is elastic, whereas Brillouin scattering and Raman scattering are inelastic.

### 2.2. Temperature Sensitivity of Raman Scattering

Raman scattering is inelastic and is produced by the combination of molecular vibration and optical phonon excitation; it can realise distributed detection of fibre temperature. When a laser pulse enters the fibre, photons interact with the inelastic collision of the medium molecules, and the photons are converted into anti-Stokes photons and Stokes photons correspondingly [23,24,25,26,27], as presented in Equation (1):

(1) $$\left\{ \begin{array}{l} hv_{s}=h(v_{0}-\Delta v)\\ hv_{a}=h(v_{0}+\Delta v) \end{array}\right.,$$

where *h* is the Planck constant; *v_a_* and *v_s_* are the Raman photon frequencies of the anti-Stokes and Stokes light; *v*_0_ is the frequency of the incident light; and Δv = 1.32 × 10^13^ Ηz is the frequency of Raman optical phonons.

The power distributions of anti-Stokes light and Stokes light on all optical fibres are as presented in Equation (2) and Equation (3):

(2) $$P_{as}=P_{0}K_{a}S_{b}v_{a}{}^{4}\frac{\exp(-h\Delta v/kT)}{1-\exp(-h\Delta v/kT)}\exp[-(\alpha_{0}+\alpha_{a})L],$$

(3) $$P_{s}=P_{0}K_{s}S_{b}v_{s}{}^{4}\frac{1}{1-\exp(-h\Delta v/kT)}\exp[-(\alpha_{0}+\alpha_{s})L],$$

where *P_as_* is the anti-Stokes optical power; *P_s_* is the Stokes optical power; *P_0_* is the optical power injected into the distributed fibre by the laser; *K_a_* and *K_s_* are anti-Stokes light and Stokes light Raman scattering section coefficients; *S_b_* is the distributed fibre backscattering factor; *k* is the Boltzmann constant; *α*_0_, *α_a_* and *α_s_* are fibre transmission loss factors of incident light, anti-Stokes light and Stokes light, respectively; and *L* is the length of the distributed fibre.

Since anti-Stokes light is more sensitive to temperature changes than Stokes light, both the single demodulation method of anti-Stokes light and the double demodulation method of anti-Stokes light and Stokes light can be used to detect the temperature distribution along the fibre [28]. By introducing the reference temperature *T*_0_, the ratio of the two signals is obtained, the attenuation of the fibre is eliminated, and the temperature distribution of the fibre can be obtained from Equation (4):

(4) $$\frac{1}{T}=\frac{1}{T_{0}}-\frac{k}{h\Delta v}\left[\ln\frac{P_{as}(T)/P_{s}(T)}{P_{as}(T_{0})/P_{s}(T_{0})}\right].$$

### 2.3. Principle of Optical Time Domain Reflection

**Figure 2:**
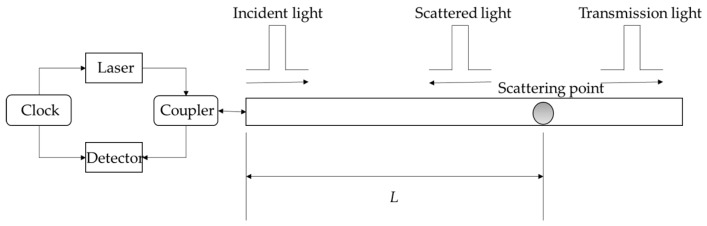
Optical time domain reflection (OTDR) working principle.

Optical time domain reflection (OTDR) is a measurement technique proposed by Barnoski in 1976. The working principle is shown in Figure 2. The clock-controlled laser emits a light pulse, which is injected into the fibre through a bidirectional coupler. Due to the non-uniformity of the medium, the light pulse will continuously generate backscattered light during transmission, and the scattered light reaches the starting position of the sensing fibre detected by the photodetector via a bidirectional coupler where it is converted into a corresponding electrical signal [29].

Assuming that the time elapsed during this process is *t*, the light pulse has a round-trip propagation path from the transmitting end to the scattering point in that time, of path length 2*l*. Therefore, the specific position of the scattering point can be obtained from Equation (5):

(5) $$l=\frac{vt}{2},$$

where *l* is the distance to the scattering point from the head end of the fibre; *v* is the propagation speed of the light pulse in the fibre; and *t* is the time elapsed from the time the probe pulse is emitted until the backscattered light is received. Thus, the scattering of distributed fibres along different longitudinal directions can be represented by the intensity of the backscattered light received by the detector at different times. When there are abnormalities such as strain, local overheating, and break-points in the optical fibre, the pulse intensity detected by the detector will undergo rapid attenuation. According to Equation (5), the abnormal point can be spatially located to realise the monitoring of optical fibre health status. 

## 3. Stability Analysis of Distributed Optical Fibres within the Transformer

### 3.1. The Selection of Optical Fibre and Insulation Characteristics Analysis

When applying distributed optical fibre sensing technology to the measurement of transformer winding temperature, the first problem to be solved is the aging and insulation of the sensing fibre inside the transformer. To ensure the stable operation of the optical fibre in the high-temperature environment of the transformer, we placed the sensing fibre of different coating layer and sheath material in the transformer oil for accelerated heat aging assessment. By comparing the electrical and physicochemical properties of transformer oil under different aging times, the influence of different materials on the thermal aging characteristics of transformer oil was analysed. Ethylene tetrafluoroethylene (ETFE) was selected as sheath layer material of the sensing fibre used in the transformer. The relevant test results show that ETFE has good mechanical and insulation properties, and can withstand temperatures above 120 °C without changes to its properties. Meanwhile, the electrical aging life of ETFE is significantly longer than that of the oil-immersed insulating paper. ETFE sheathing material has little effect on the overall insulation performance of transformer oil, and can slow down the aging process of oil and high temperature on the acrylate coating inside the fibre. It is therefore an ideal distributed fibre sheathing material [30,31].

### 3.2. The Design of Optical Fibre Composite Winding

**Figure 3:**
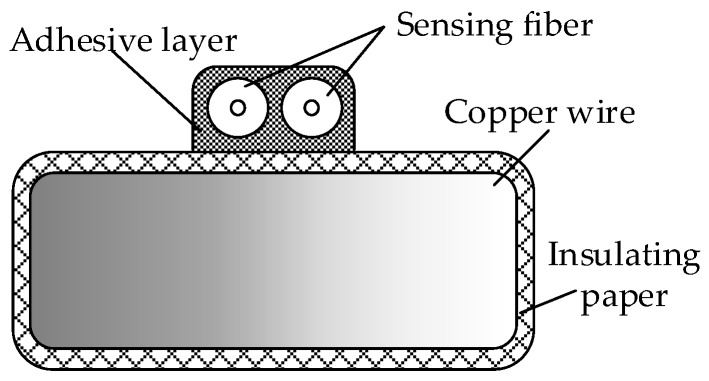
Cross-sectional view of fibre composite winding wire.

The temperature sensing was realised by attaching the optical fibre to the outermost wire surface of the transformer winding. Distributed optical fibres were fixed onto the surface of the wire using an adhesive (Figure 3). A pulley guiding device was used to wrap two layers of insulating paper around the conductor in the process of wrapping the winding copper wire to insulate the paper, thus avoiding direct contact between the fibre and the wire. We placed the tightly wound fibre on the wider side of the winding and secured the distributed fibre to the winding surface with adhesive. This arrangement does not change the winding structure, but also means that the distributed fibre remains unaffected by external oil flow, vibration and inter-turning force. When the transformer is in normal operation or local overheating occurs, the fibre senses the change in winding temperature and heats synchronously with the winding. The winding state can be judged by detecting the change in the Raman scattering signal from the fibre.

The adhesive selected was a 7102 epoxy resin adhesive. Epoxy resin adhesive has excellent bonding properties to common materials. It can be cured at room temperature. The material parameters are shown in Table 1. The relevant test results show that the epoxy resin underwent no obvious shape change under typical transformer working conditions, and was compatible with the transformer [32,33]. 

### 3.3. The Effect of Optical Fibre on Electric Field Distribution in Oil

Since distributed fibres are composed of multiple dielectrics, they may have an effect on the electric field distribution in the oil. For this study, a 110 kV transformer low-voltage winding wire was modelled via COMSOL Multiphysics (5.4, COMSOL AB, Stockholm, Sweden) to simulate the influence of the fibre on the internal electric field of the transformer under AC steady-state conditions.

**Figure 4:**
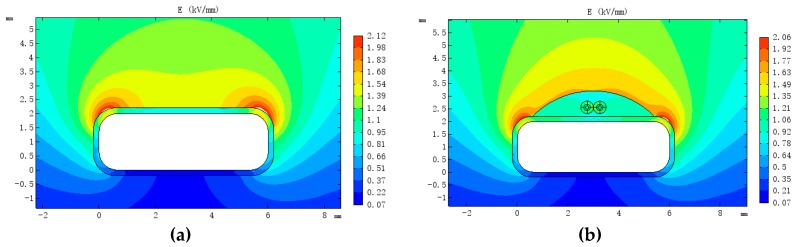

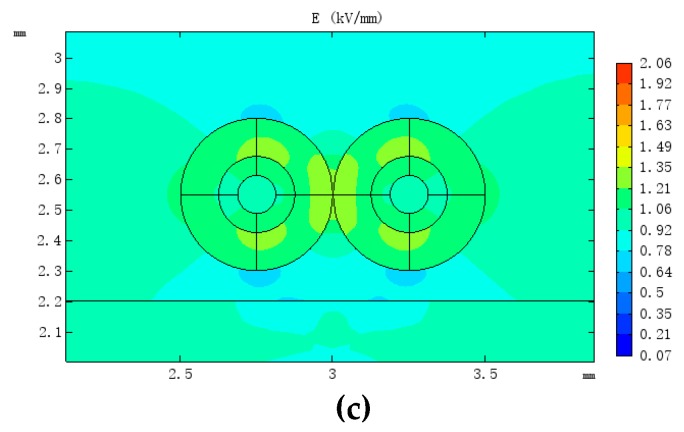
(**a**) Electrical field distribution of original conductor; (**b**) electrical field distribution of optical fibre composite conductor and (**c**) electrical field distribution in the optical fibre.

**Figure 5:**
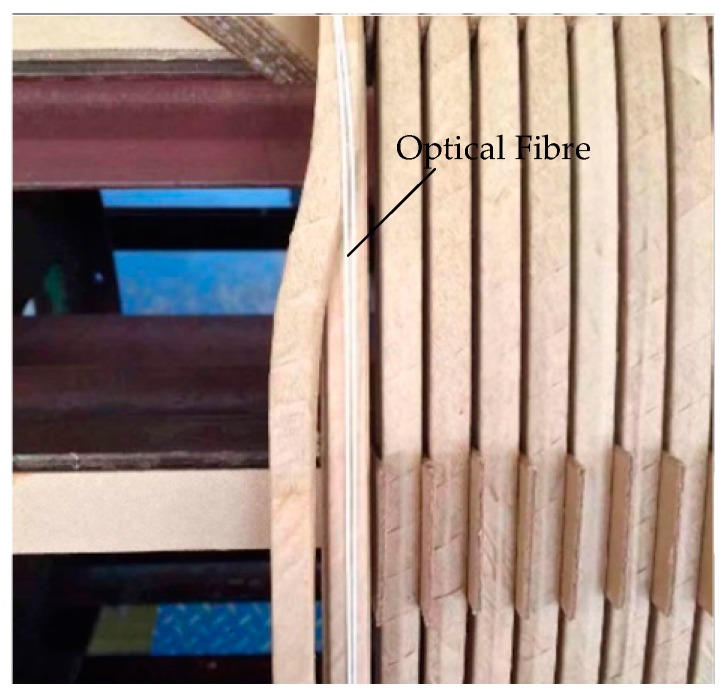
Transposition between cakes.

A two-dimensional simulation model of the fibre composite wire was established (Figure 4). The simulation parameters are listed in Table 2. The low-voltage winding had a voltage rating of 10.5 kV, a wire width of 6 mm, a height of 2 mm, a fillet radius of 0.65 mm and an insulating paper with a thickness of 0.2 mm on the surface. The optical fibre had a three-layer structure, including a core with diameter of 0.125 mm, a coating layer with thickness of 0.0625 mm and a sheathing layer with thickness of 0.125 mm. The depth of epoxy resin was 1 mm and the voltage across the wire was set to 10 kV. The variation of the electrical field distribution of the wire before and after the installation of the fibre was simulated and analysed (Figure 5).

Figure 4a,b shows that the maximum electrical field along the wire before installation was 1.98 kV/mm, and that after fibre installation it was 1.92 kV/mm (both at the corners of the wire). The maximum electrical field between the epoxy resin and the transformer oil was 1.63 kV/mm. The internal electrical field distribution around the fibre is shown in Figure 4c, and the maximum (1.21 kV/mm) is located in the sheath layer. It can be seen that although distributed optical fibre changes the original electric field distribution of the wire, it is not the cause of the increase in peak electrical field strength.

**Figure 6:**
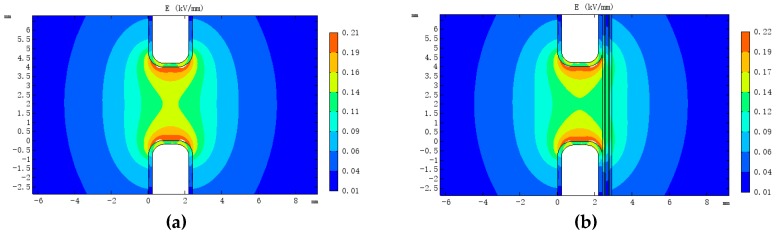
(**a**) Original electric field distribution between cakes and (**b**) electrical field distribution between cakes after installation of optical fibres.

For continuous transformer windings (Figure 5), some fibres are suspended in the oil as the fibre extends from the previous cake to the next. To explore the distortion of the electrical field between the cakes in this case, a two-dimensional model of the outermost two wires of the wire cake was established, and the influence of the fibre on the electrical field distribution between the cakes was analysed. The distance between the cakes was 5 mm, and the voltage across the low-voltage winding cakes was 700 V. The electrical field distribution between the cakes after fibre installation is shown in Figure 6.

The simulation results show that the maximum electrical field between the cakes after installation of the fibre was 0.19 kV/mm, occurring at the junction between the insulating paper, transformer oil and fibre sheath layer. The maximum value was increased by 1.03% compared with that before fibre installation. The maximum internal field strength around the sensing fibre was 0.14 kV/mm, as found in the sheath layer.

### 3.4. The Heat Transfer between Optical Fibre and Wire

**Figure 7:**
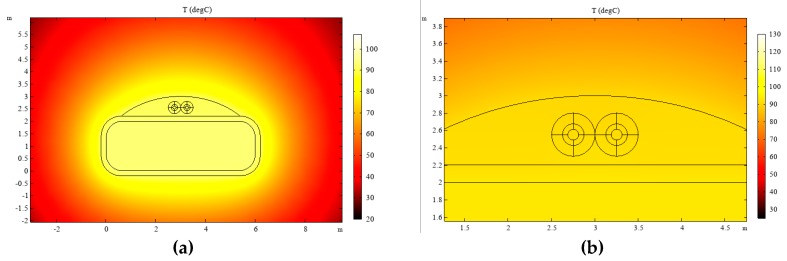
(**a**) Temperature distribution of optical fibre composite conductor and (**b**) temperature distribution in the optical fibre.

To study the efficiency of temperature transfer between optical fibre and wire, and to correct the results of fibre measurement, based on the optical fibre composite wire model established above, the temperature field distribution under steady-state conditions was simulated via COMSOL Multiphysics. The density, thermal conductivity and constant pressure heat capacity of each material are shown in Table 3. The ambient temperature was set to 20 °C, the heat loss rate of the wire was 40 W and the convective heat transfer coefficient was 310. The temperature field distribution of the wire after fibre installation is shown in Figure 7.

The simulation results show that the temperature of the wire after installation of the fibre was 92.96 °C, whereas the internal temperature of the fibre was 92.44 °C and the distribution of heat was even. The efficiency of heat transfer between the distributed optical fibre and the wire was 99.44%. This shows that the adhesive fibre can sense changes in wire temperature and meet transformer winding temperature measurement requirements.

### 3.5. Power Frequency Resistance Testing

**Figure 8:**
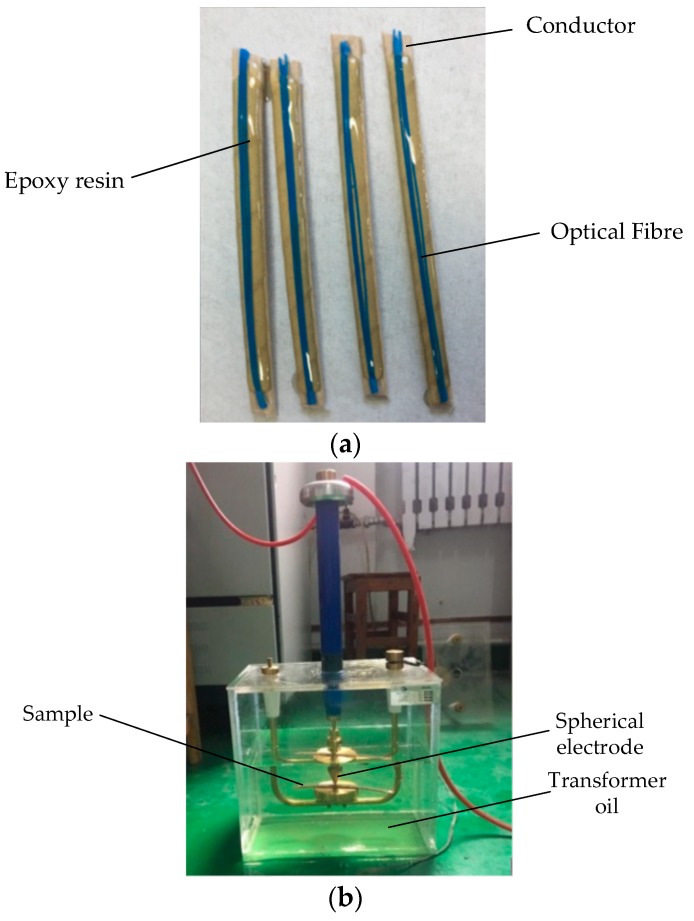
(**a**) Sample preparation and (**b**) breakdown voltage measuring device.

We used epoxy resin to attach the fibre to the winding surface (Figure 8a): after curing at room temperature for 24 h, it was immersed in transformer oil which had been vacuum-dried for 24 h, and immersed under vacuum at 40 °C for 24 h, so that the insulating oil filled the gap in the insulating paper and interacted with the epoxy resin. After impregnation, a ball-type electrode was used for the breakdown test (Figure 8b). The boosting method referred to IEC 60243-1:1998, which specified a starting voltage *U*_0_ of 10 kV, applied in increments of 2 kV. The voltage applied at each stage was maintained for 20 s until the sample broke down or flashed.

**Figure 9:**
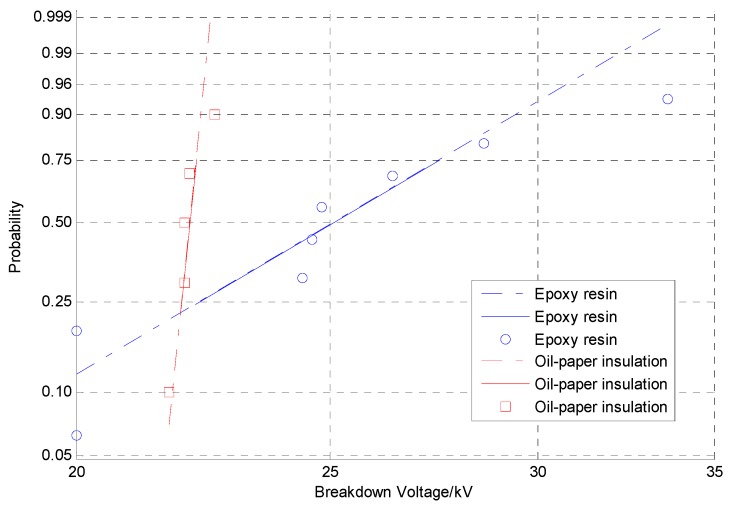
Weibull probability plot.

According to the test data, the results of multiple breakdown tests were evaluated by the two-parameter Weibull distribution. As shown in Figure 9, the 63.2% breakdown voltage of the 7102 epoxy resin (26.3 kV) was higher than that of the oil-paper insulation gap (22.1 kV) at an equivalent distance. Therefore, it can be used for the binding and fixing of the internal fibres of the transformer.

## 4. Distributed Temperature Detection in a Transformer Winding

### 4.1. Building the Test Platform

**Figure 10:**
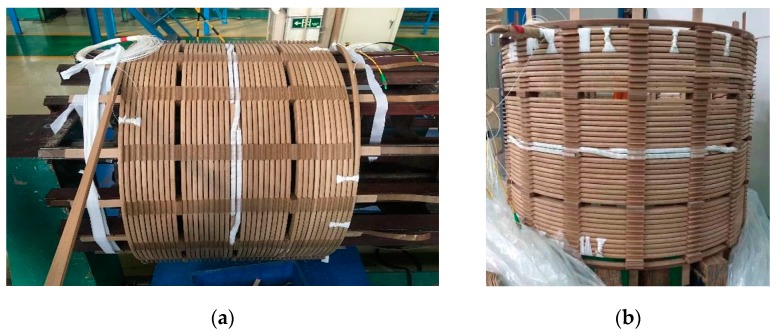
(**a**) The installation of fibre-optic composite transformer winding model and (**b**) the completed transformer winding model.

The winding model was made based on the size of a 31.5 MVA 110 kV transformer low-voltage winding. Eight pieces of wires were wound in parallel and the outermost wire was replaced by the fibre composite wire shown in Figure 3. Finally, a winding model with an outer diameter of 700 mm and a total of 40 cakes and length of approximately 90 m was produced, as shown in Figure 10. To eliminate measurement error caused by the head-end blind area and tail-end reflection, 20 m tail fibres were connected to the beginning and end of the model.

Raman optical time domain reflectometry (ROTDR) is a sensing system based on spontaneous Raman scattering. It has the advantages of single-ended incidence and distributed measurement, which can realise distributed detection of winding temperature. ROTDR uses the distributed optical fibre temperature monitoring system produced by Weihai Beiyang Optoelectronic Info-Tech Co. Ltd. (Weihai, China). The system has the advantages of high precision and large temperature range. Generally, the time required to measure the temperature of a fibre is between 2 and 10 s, which satisfied the requirements of transformer temperature measurement and winding hot-spot temperature positioning. The instrument parameter settings are listed in Table 4.

### 4.2. Temperature Sensing Performance Test

**Figure 11:**
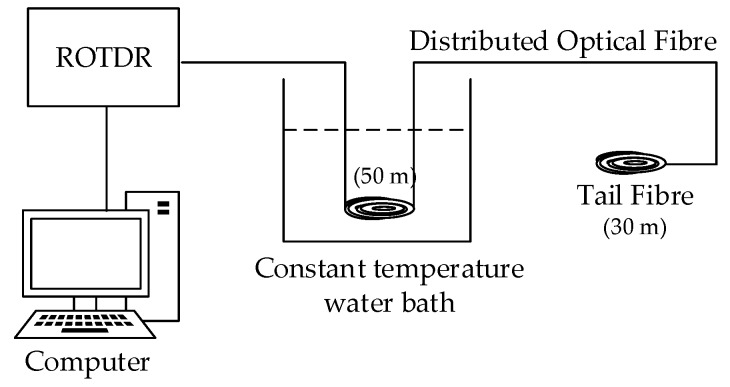
Distributed optical fibre temperature calibration system.

**Figure 12:**
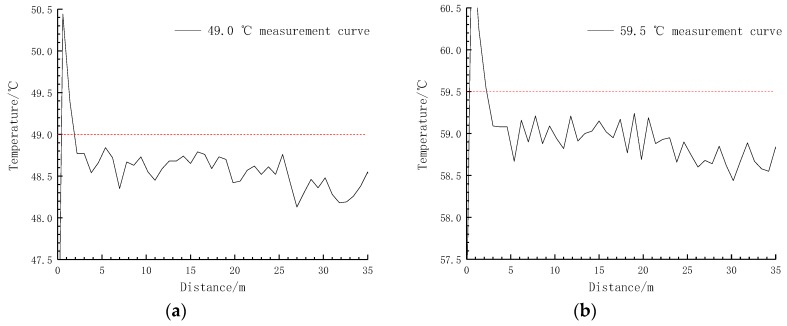

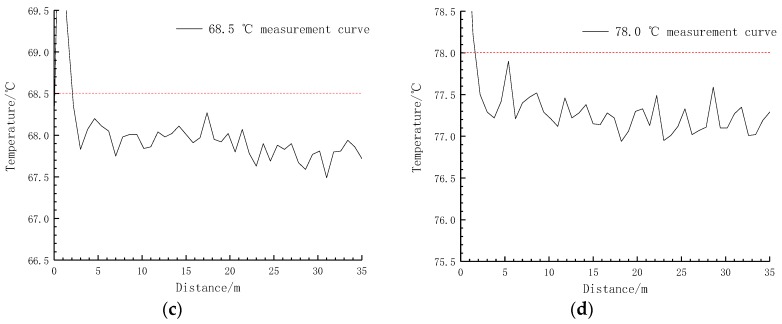
Temperature measurement data: (**a**) 49.0 °C measurement curve; (**b**) 59.5 °C measurement curve; (**c**) 68.5 °C measurement curve and (**d**) 78.0 °C measurement curve.

To verify that the transformer winding of the externally distributed fibre had good temperature sensing performance, a distributed fibre temperature calibration platform was built in the laboratory, and the temperature calibration experiment was performed on the ETFE optical fibre (Figure 11). The specific calibration process was as follows: take 100 m of calibrated ETFE optical fibre, connect the first end to the ROTDR system via jumper wire, take 50 m of loose fibre, form it and place it into a constant temperature water bath with a temperature control accuracy of ±0.005 °C. The working temperature of the bath was set to 49.0 °C, 59.5 °C, 68.5 °C, and 78.0 °C, respectively. After the temperature of the water bath had stabilised, it was measured by the distributed temperature measuring system (Figure 12).

It can be seen from Figure 12 that the ROTDR system measurement error is less than 1 °C, indicating that the distributed optical fibre measurement method is feasible. 

### 4.3. Winding Model Test

The temperature sensing performance test proves that the transformer windings with distributed fibres offered good temperature sensing performance. To verify the feasibility and reliability of the distributed fibre-based transformer winding temperature measurement method, a test platform consisting of fibre composite winding models and a distributed optical fibre temperature measurement system based on Raman scattering was built.

**Figure 13:**
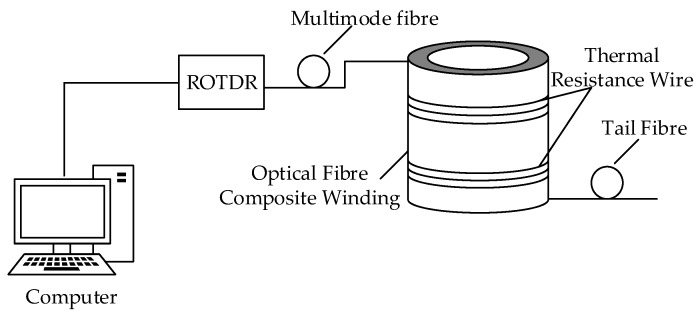
Temperature measurement system using optical fibre.

First, the temperature of the winding model at room temperature was measured by a distributed optical fibre temperature measurement system and a standard thermocouple, the latter showing that the room temperature was at 29.5 °C. Second, adding a thermal resistance wire to the gap between the winding cakes and pressurising the wire through the voltage regulator achieved the purpose of heating the winding cakes (Figure 13).

**Figure 14:**
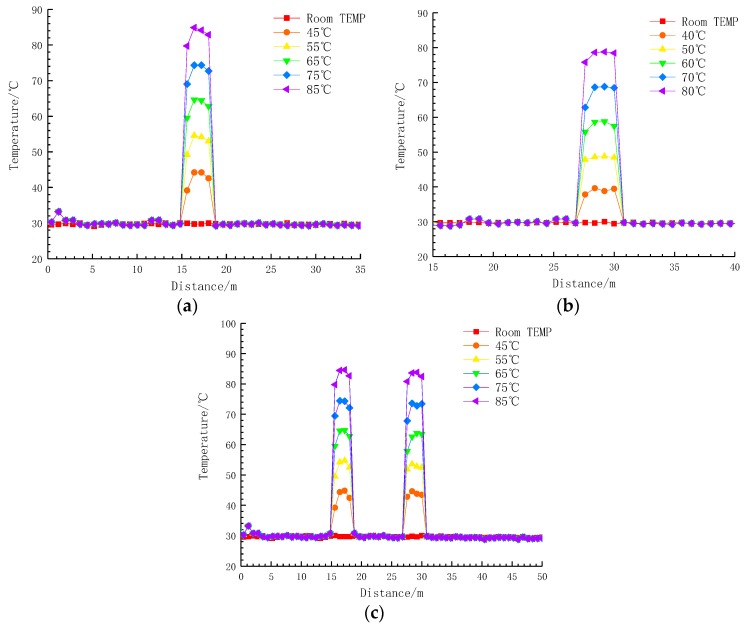
(**a**) The 8th cake heating up; (**b**) the 13th cake heating up and (**c**) the 8th and 13th cakes heating up.

Using this method, the 8th and 13th cakes were heated, and the temperature of the winding model was measured by a distributed optical fibre temperature measuring system and a standard thermocouple, respectively. The temperature distribution of the winding model at five different measurement points is shown in Figure 14. Figure 14a–c shows the temperature distribution on the 8th, 13th and both cakes. The test was carried out after the temperature had stabilised. Then the temperature distribution curve measured by the distributed optical fibre temperature measuring system was compared with that measured at room temperature.

To analyse the measurement results more intuitively, the actual and measured positions of the winding temperature rise, the temperature measured by the distributed optical fibre and the standard thermocouple were compared, based on the aforementioned winding model parameters. The results are summarised in Table 5 and Table 6.

According to the local hot-spot detection results, the areas exhibiting a temperature rise, as measured by the distributed optical fibre, were larger than the actual areas heated on these windings. This is because the sampling resolution of the distributed optical fibre temperature measurement system is between 0.4 and 0.8 m, and the measured data represent an average temperature between two adjacent sampling points. Therefore, there is a certain response transition distance before, and after, the warmer area on the winding. From the results, the error between the measured distance and the actual distance remained within 1 m.

According to the measurement of hot-spot temperatures on the winding, the error between the temperature measurement result and the standard thermocouple measurement result is limited to 1 °C, which indicates that the temperature measurement method returned a higher accuracy.

## 5. Conclusions

A distributed optical fibre sensing technology based on Raman scattering to measure the temperature of transformer windings was proposed, the optical fibre composite winding was designed and a distributed optical fibre temperature measurement platform was built. The temperature sensing performance test and the winding model test were conducted. The conclusions are as follows:

(1) The transformer winding composite model with distributed fibre can transmit real-time temperature information of the winding, and the temperature information measured by ROTDR reflects the change in the state of the winding;
(2) The temperature sensing performance test results verify the feasibility of the distributed optical fibre sensing technology in detecting the temperature field distribution of a transformer winding, and the temperature measurement result error is limited to 1 °C, which satisfies the requirements of transformer winding temperature measurement;
(3) Winding model test results show that the transformer winding temperature measurement method based on distributed optical fibre sensing can realise the measurement of winding temperature and the location of an abnormal local temperature rise therein. The method offered high positioning accuracy with a positioning error of less than 1 m;
(4) The characteristics of distributed optical fibres determine their potential for use in the online monitoring of transformer status; this can overcome the shortcomings of traditional detection methods. In subsequent research, the relationship between the variation of Raman scattering and the local hot-spot distribution on such windings will be investigated to provide more accurate status information about windings for engineers who are responsible for the maintenance of transformers.

## Figures and Tables

**Table 1:** Parameters of epoxy resin adhesive.

Parameter	Value
Basic cured time/min	5–10
Fully cured time/h	3–7
Temperature range/°C	−40 to +130
Bond strength/MPa	10.5

**Table 2:** Model parameters for electrical field simulation.

Material	Transformer Oil	Insulating Paper	Adhesive Layer	Core	Coating Layer	Sheath Layer
Relative dielectric constant	2.1	3.6	3.0	4.1	2.9	2.6

**Table 3:** Model parameters for temperature field simulation.

Material	Transformer Oil	Insulating Paper	Adhesive Layer	Core	Coating Layer	Sheath Layer
Density (kg·m^−3^)	890	1150	1000	2300	1150	1000
Thermal conductivity (W·(m·K)^−1^)	0.13	0.25	2.2	7.6	0.08	0.1
Constant pressure heat capacity (J·(kg·K)^−1^)	1857	1929	1000	966	1970	385

**Table 4:** ROTDR parameter settings.

Parameter	Value
Sampling interval/m	0.4–0.8
Spatial resolution/m	1
Temperature accuracy/°C	±1
Temperature resolution/°C	≤0.5
Temperature range/°C	−190.0 to +700.0
Maximum monitoring distance/m	2000.0
Measurement time/s	2.0–10.0

**Table 5:** Comparison of winding hot-spot positioning results.

Test Results	First Measurement	Second Measurement	Third Measurement	
Heating position/cake	8	13	8,13	
Actual position/m	15.75–18.00	27.00–29.25	15.75–18.00	27.00–29.25
Measurement position/m	15.32–18.24	26.64–30.06	14.91–18.73	26.23–30.32
Result	Accurate	Accurate	Accurate	

**Table 6:** Comparison of winding hot-spot temperature measurement results.

Test Results	First Measurement					Second Measurement					Third Measurement				
Heating position/cake	8					13					13				
Thermocouple temperature/°C	45.0	55.0	65.0	75.0	85.0	40.0	50.0	60.0	70.0	80.0	45.0	55.0	65.0	75.0	85.0
Measurement temperature/°C	44.5	54.1	64.3	74.4	84.1	39.2	49.5	59.5	69.2	79.4	44.5	54.2	64.3	74.1	84.4
Result	Accurate					Accurate					Accurate				
